# Intermittent auscultation fetal monitoring practice in different UK birth settings: a cross-sectional survey

**DOI:** 10.1186/s12884-025-07514-2

**Published:** 2025-04-14

**Authors:** Megan Douthwaite, Alessandra Morelli, Sara Kenyon, Julia Sanders, Rachel Rowe

**Affiliations:** 1 National Perinatal Epidemiology Unit, Nuffield Department of Population Health, University of Oxford, Old Road Campus, Oxford, OX3 7LF UK; 2 School of Health Sciences, University of Birmingham, Edgbaston, Birmingham, B15 2TT UK; 3 School of Healthcare Sciences, Heath Park Campus, Cardiff University, Cardiff, CF14 4XN UK

**Keywords:** Intermittent auscultation, Midwifery, Fetal heart rate, Fetal monitoring, Intrapartum, United Kingdom

## Abstract

**Background:**

Intrapartum fetal heart rate monitoring is used to assess fetal wellbeing throughout labour. The interpretation of fetal heart rate patterns during labour informs decisions about clinical management and intervention. The World Health Organisation and other professional health care bodies recommend intermittent auscultation (IA) for monitoring the fetal heart rate for women with uncomplicated labour. Despite this there is little research on how IA is carried out in practice. This study aimed to describe IA practice across different birth settings in the United Kingdom (UK).

**Methods:**

We conducted an online cross-sectional survey between November 2022 and February 2023. The survey explored whether local guidance on IA was in place; the frequency of IA training and competency assessment and packages used; content and frequency of IA audits; access to and use of IA devices; fetal heart rate counting methods used; and use of ‘fresh ears’. We invited respondents from 205 alongside and freestanding midwifery units, and 33 obstetric units in National Health Service (NHS) organisations without midwifery units, from 140 NHS organisations across the UK. Descriptive statistics were used to analyse responses about IA practice by birth setting.

**Results:**

One hundred and seventy-four units (73%) responded from 119 NHS organisations. Most (91%) had local IA guidance in place for midwifery or obstetric led care, or both. While most maternity units (58%) required midwives to undertake annual IA training and competency assessments, 18% did not. A third of units reported an annual IA audit, but 67% of units had no set frequency or did not know the timing of their unit IA audit. At least six different methods for counting the fetal heart rate were reported, with 45% using some form of ‘Intelligent IA’ counting method. Just under half of units reported implementing ’fresh ears‘ for IA.

**Conclusions:**

This was the first national survey of IA practice in the UK, and provides evidence of widespread variation in practice. Further investigations would be helpful to better understand why certain practices are followed or not, and the rationale behind these decisions in a clinical setting. Evidence to inform IA best practice is urgently needed.

**Supplementary Information:**

The online version contains supplementary material available at 10.1186/s12884-025-07514-2.

## Introduction

Intrapartum fetal heart rate monitoring is used to assess fetal wellbeing throughout labour and contributes to decisions about clinical management and intervention [1]. The World Health Organization (WHO) and other professional healthcare bodies [2–5], including the United Kingdom’s (UK) National Institute for Health and Care Excellence (NICE) [6] recommend the use of intermittent auscultation (IA) to monitor the fetal heart rate during uncomplicated labour in healthy women with straightforward pregnancies. In the UK, this is an essential component of intrapartum midwifery care and involves listening to and counting the fetal heart rate for short specified amounts of time at specified intervals using a Pinard stethoscope or handheld Doppler ultrasound device [2, 6].

For women who are at ‘low risk’ of intrapartum complications at labour onset, IA is recommended over electronic fetal monitoring (EFM) using cardiotocography (CTG) [2, 3, 5, 7–15]. Performing a short ‘admission CTG’ at labour presentation in low risk women increases the chance of intervention during labour or birth, including the use of continuous CTG in labour, without clear evidence of improved neonatal outcomes [7, 11, 16]. While some evidence suggests CTG has been associated with a reduction in neonatal seizures [2, 10, 17], the use of continuous CTG in low risk women is associated with increased rates instrumental and caesarean birth, again with no clear evidence of other improved outcomes [3, 6, 7, 11]. IA offers women greater choices of position and freedom of movement [2]; and an enhanced sense of autonomy during the birth process [5]; in low-resource settings it may be more practical and cost-effective [5, 18].

UK national guidance recommends that women should have access to four types of birth setting: hospital obstetric unit (OU); alongside midwifery unit (AMU), located on the same site as an OU; freestanding midwifery unit (FMU), geographically separate from an OU; and home. For women at low risk of complications, planning birth in a midwifery unit (MU) is associated with higher rates of spontaneous vaginal birth and lower rates of intervention than a planned birth in an OU, without any detrimental impact on neonatal outcomes. In England in 2015, the most recent year for which national data are available, 14% of births took place in midwifery units (AMU or FMU) [19] where IA is used to monitor fetal wellbeing. IA is also used in planned home births, estimated at 0.2% of births in Northern Ireland to 2% in England and Wales in 2021 [20, 21]. The extent to which IA is used for fetal monitoring in low risk women admitted to OUs is unknown.

National guidance on the timing, frequency and duration of IA monitoring dates back to 2001 in the UK [15], and several updates have been made since [6, 22]. Multiple reports and enquiries have repeatedly identified sub-optimal monitoring and responses to fetal heart rate changes - using both IA and CTG - as contributing to preventable adverse outcomes [23–26]. Improvement initiatives have recommended better IA training and called for more research about IA training and competency assessment [27]. Guidance from the Royal College of Obstetricians and Gynaecologists (RCOG) in 2001 recommended annual training with assessment in IA and EFM [15], and since then best practice guidance for the National Health Service (NHS) in England has recommended that all midwives undertake annual training and competency assessment in IA [28]. Guidance about the principles that should be followed has been developed, but there remains little detail on the content of training or competency assessment. Since 2019 guidance has also recommended the use of a buddy system whereby another healthcare practitioner provides a ‘fresh ears’ interpretation of IA [29, 30].

There remains a lack of evidence about the ideal IA monitoring device; the optimal timing, frequency and duration of IA [2]; or any descriptive information of how midwives carry out IA in practice; and any practical, organisational or systems-level barriers and facilitators to IA practice and documentation. Evidence from Norway found that a fifth of units did not have local criteria for when to apply different fetal monitoring methods [11], and revealed some variation in practice and deviation from clinical recommendations, namely the use of intermittent CTG monitoring for women with uncomplicated, low risk labour [31]. Research into how midwives practice IA in the UK is overdue. As part of the Listen2Baby study, investigating the practice of IA in the UK with the aim of improving the quality and safety of IA, the survey reported here aimed to describe IA practice across different birth settings in the UK. We explored whether local IA guidance was in place; the frequency of IA training and competency assessment and packages followed; the content and frequency of IA audits; access to and use of IA devices; fetal heart rate counting methods used; and the use of the buddy system for ‘fresh ears’.

## Methods

### Study design and data collection

We conducted an online cross-sectional survey between 10^th^ November 2022 and 3^rd^ February 2023. Adopting an approach used in previous surveys, we used the UK Midwifery Study System (UKMidSS), a national research infrastructure supporting observational studies and surveys of practice in UK MUs [32], as the sample frame. We invited all 205 MUs contributing to UKMidSS studies at the time of the survey to take part (representing more than 90% of all UK MUs at the time of the survey). We also invited 33 OUs in NHS organisations (Trusts or Health Boards) without MUs to participate, using the list of obstetric units available from the National Maternity & Perinatal Audit Organisational Report 2019 [33] as the sample frame.

Email invitations were sent to UKMidSS midwife ‘reporters’ in each MU, and Heads of Midwifery and Fetal Monitoring Lead Midwives in the OUs. They were asked to respond on behalf of their unit by completing a short online survey about IA device availability and IA practice in their unit and NHS organisation. The invite included a hyperlink to allow individualised access to the survey in the secure online platform LimeSurvey [34]. The survey was available for three months and closed thereafter. Up to nine reminder emails were sent to non-respondents.

UKMidSS ‘reporters’ are typically nominated for their role by the Head or Director of Midwifery or MU manager. Most are midwives who work in or have managerial responsibility for the MU, while a small number are research midwives [35]. In several MUs there is more than one UKMidSS ‘reporter’. Invitations were sent to all reporters and where more than one response per unit was received, we analysed the most complete and prioritised the first received.

Data concerning the type of unit (AMU/FMU) and nation of the UK (and NHS region within England) in which the unit was located were extracted from the UKMidSS administrative system.

### The questionnaire

Two versions of the questionnaire, one for MUs and the second for OUs in NHS organisations without MUs, were developed by RR, JS and SK specifically for this study (Supplementary Material 1 & 2). Both covered the same topics, and comprised single and multiple-choice (tick all that apply) questions, with some open-ended questions for free text responses (Supplementary Material 3). All respondents were asked about 1) IA policy; 2) IA training and competency assessment; 3) IA practice audits; 4) availability of different types of IA devices and devices typically used; and 5) about IA practice , including the method used to count the fetal heart rate, use of a buddy system, and short admission CTG; and lastly the purchase of IA devices. Questions were also asked about IA practice in other types of birth setting in their NHS organisation, including home births and OUs. MU respondents were asked about IA practice in home birth settings and in OUs in their NHS organisation. OUs in NHS organisations without MUs were asked about IA practice in home birth settings. The MU questionnaire was piloted with six UKMidSS reporters to verify the clarity of the questions and terminology used.

### Data management

Several composite variables were created to explore multiple response questions about 1) IA device availability; 2) IA device use; 3) methods to count the fetal heart; and 4) compulsory training and assessment packages. For these variables, categories with fewer than six responses in total were recoded as “other”, and the detail presented in a footnote below the table/figure. For the ‘methods of counting’ question, where respondents indicated there was ‘no counting method required’ and reported that one or more specific counting methods were used, these latter methods were used for analysis.

Respondents were asked to select the training and assessment packages they were required to undertake from a pre-assigned list of responses (see Supplementary Table 1 for detail), and to provide detail using free text boxes on *in-house* and *other* compulsory IA training and competency assessments. Where possible free text responses were recoded to create categorical variables (e.g. K2 training) detailing specific training and assessment packages.

### Data analysis

To assess response bias, Fisher's exact tests were used to compare responses by unit type (AMU/FMU), and by nation (England, Scotland, Wales & Northern Ireland).

We present numbers and percentages for categorical data by type of unit: AMU; FMU; and OUs in NHS organisations without MUs. We present data about whether NHS organisations have local IA guidance in place (yes/no); the frequency of IA training and competency assessments, and IA practice audits; availability and typical use of IA devices; performance of a short admission CTG; purchase of Dopplers; and the practice of fresh ears and methods of counting the fetal heart rate. We used Fisher’s exact tests to explore significant differences. In the case of multiple-choice questions, we present frequency distributions of single responses, as well as the distribution of multiple combinations of responses. Where appropriate selected free-text responses are reported.

Responses to questions that asked about IA practice in MUs were analysed at the unit level. Responses to questions about organisation-level practice - whether local guidance was in place, the purchase of devices, and the use of IA devices for home births and in OUs - were analysed at NHS organisation level. When NHS organisations had more than one responding MU, we prioritised responses from AMUs about IA practice in OUs, and from FMUs about IA practice in home births, with the assumption that AMU midwives were more likely to know about practice in the adjacent OU, and that FMU midwives were more likely to know about practice at homebirths. In the absence of responses from prioritised units within NHS organisations, we included responses from either FMUs (for OUs) or AMUs (for homebirths). Where more than one unit of the same type responded per NHS organisation, we combined unit responses to create one response per NHS organisation. For example, in the case of three AMUs responding from the same NHS organisation, we prioritised “yes” responses over “no”, even if two of three units had responded “no”.

There were few missing values and these are shown in the tables. All analyses were conducted using STATA 17 [36].

### Patient and public involvement (PPI)

The Listen2Baby co-investigator group includes two lay members who represented the views of pregnant women and their families throughout the design, conduct and interpretation of this study.

### Consent to participate

Informed consent was obtained from all participants.

## Results

### Response

**Figure 1:**
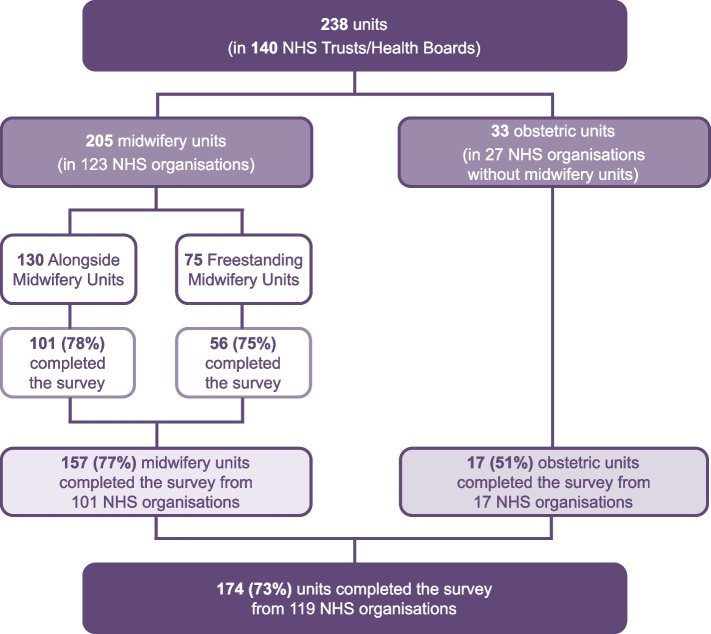
Study flow chart

Two hundred thirty-eight units from 140 NHS organisations across all four nations of the UK were invited to participate in the survey (Figure 1). In total, 174 units (73% of those contacted) from 119 NHS organisations (85%) responded. The response rates varied significantly by type of unit (p≤0.013) and nation (p≤0.002). Over 75% of AMUs and FMUs responded, whereas 52% of OUs in NHS organisations without MUs responded. The response rate ranged from 100% in Wales to 56% in Northern Ireland. Reflecting the distribution and type of units across the UK, most responses included in these analyses were from units in England (73%), and most (*n*=101) were AMUs (see Table 1).

**Table 1:** Response rate by type of unit, overall and by nation of the UK

	**Alongside Midwifery Units (AMUs)**			**Freestanding Midwifery Units (FMUs)**			**Obstetric units in NHS organisations without MUs**			**Total**		
**Country**	**Contacted (N)**	**Responded (n)**	**%**	**Contacted (N)**	**Responded (n)**	**%**	**Contacted (N)**	**Responded (n)**	**%**	**Contacted (N)**	**Responded (n)**	**%**
England	107	82	76.6	46	31	68.9	30	14	46.7	183	127	69.3
Wales	10	10	100.0	11	11	100.0	0	0	0	21	21	100.0
Northern Ireland	6	3	50.0	3	2	66.7	0	0	0	9	5	55.6
Scotland	7	6	85.7	15	12	80.0	3	3	100.0	24	21	84.0
Total	130	101	76.9	75	56	75.7	33	17	51.5	238	174	73.1

### IA local guidance in place

Most NHS organisations, with or without MUs, had local guidance on IA in place for midwifery led care or OUs (see Table 2). Overall 75% (*N*=110) of NHS organisations included in this analysis reported having local IA guidance in place for both midwifery led care and OUs (data not shown), and 8% (*n*=9) reported that there was no guidance on IA use in either setting. Seventeen NHS organisations with MUs (16%) and two without MUs (2%) reported having local IA guidance in place for midwifery led care only, and not for their OU. Twenty-three units provided qualitative comments about local IA guidelines, adding that guidelines focus mainly on “risk status” of the pregnant woman rather than the birth setting.

*“Women [in the] OU that are suitable for IA have IA. Women [in the] OU who meet the criteria for CTG have a CTG. IA guidance is only for straightforward pregnancies meeting the criteria for the OU or MU / FMU”. (AMU, England)*

*“We aim for all women who are considered low risk in pregnancy and birth (midwife led care) to birth outside of the OU. When women choose to birth on the OU, they follow the MLC pathway, which means IIA and no CTGs. If they need to move off the MLC pathway due to requesting an epidural or other factors, then they would follow the OU pathway and have CTG/ ST analysis”. (FMU, Wales).*

**Table 2:** Percent of NHS organisations with local IA guidance for midwifery led care settings and obstetric units

	**NHS organisations with midwifery units (*N*=102)**		**NHS organisations without midwifery units (*N*=17)**		**Total (*N*=119)**		*p*-value*
	**n**	**%**	**n**	**%**	**n**	**%**	
**Local IA guidance in place for**							
Midwifery led settings (MU & home births)	85	90.4	14	87.5	99	90.0	0.718
Obstetric Units	72	76.6	12	75.0	84	76.4	0.890
No guidance in place for either setting (OU, MU or homebirth)	7	7.5	2	12.5	9	8.2	0.849
*missing*	*(8)*		*(1)*		*(9)*		

### IA training and competency assessment

Compulsory IA training and competency assessments for midwifery staff were reported by 56% of AMUs (*n*=57), 59% of FMUs (*n*=33), and 65% (*n*=11) of OUs in organisations without MUs. A further 34% of AMUs and 29% of FMUs reported compulsory IA training only (without assessment). Overall, 12% (*n*=20) of units reported that there was no compulsory IA training or competency assessment for midwifery staff (Table 3). While most units that reported compulsory IA training and/or competency assessment reported that it was an annual requirement, 18% of all units reported that it was not an annual requirement, or that there was no such mandated IA training and competency assessment.

**Table 3:** Frequency and type of mandatory IA training and assessment by type of unit

	**AMU (*N*=101)**		**FMU (*N*=56)**		**OUs in NHS organisations without MUs (*N*=17)**		**Total (*N*=174)**		*p*-value*
	**n**	**%**	**n**	**%**	**n**	**%**	**n**	**%**	
**Mandatory IA training & competency assessment**									
Yes, training & competency assessment	57	56.4	33	58.9	11	64.7	101	58.1	
Yes, training only	34	33.7	16	28.6	3	17.7	53	30.5	
No training or assessment required	10	9.9	7	12.5	3	17.7	20	11.5	0.650
**Frequency of IA training & competency assessment**									
Annual - training & assessment	55	54.5	31	55.4	11	64.7	97	55.8	
Annual - training only	31	30.7	12	21.4	3	17.7	46	26.4	
Every other year - training & assessment	2	2.0	2	3.6	0	0.0	4	2.3	
Every other year - training only	1	1.0	3	5.4	0	0.0	4	2.3	
No set frequency - training & assessment	2	2.0	1	1.8	0	0.0	3	1.7	
No training or assessment required	10	9.9	7	12.5	3	17.7	20	11.5	0.722
**Required IA training and competency assessment package**									
In-house training (content unspecified)	38	37.6	8	14.3	5	29.4	51	29.3	
K2	23	22.8	10	17.9	3	17.7	36	20.7	
NHS e-learning for healthcare ‘IIA'^a^ training and competency assessment only.	8	7.9	20	35.1	2	11.1	30	17.2	
NHS e-learning for healthcare ‘IIA’ training and competency assessment **PLUS** in-house training (content unspecified)	8	7.9	5	8.9	3	17.7	16	9.2	
NHS e-learning for healthcare ‘IIA’ training **PLUS** in-house competency assessment	7	6.9	4	7.1	1	5.9	12	6.9	
Other combinations of training and competency assessment	7	6.9	2	3.6	0	0.0	9	5.2	
No training or assessment required	10	9.9	7	12.5	3	17.7	20	11.5	<0.001

^*^Fischer's exact test

^a^Intelligent Intermittent Auscultation

The type of mandatory IA training and assessment undertaken varied by type of unit. The most common options among AMUs and OUs were *in-house* (content not specified) training and competency assessment, or a package provided by *K2 Medical Systems* (Table 3). Just over one-third of FMUs (35%) reported sole use of the NHS e-learning for healthcare ‘Intelligent Intermittent Auscultation (IIA)’ training and competency assessment, compared with 8% of AMUs and 11% of OUs. Over half of FMUs (*n*=29, 52%) and 22% (*n*=23) of AMUs reported using part or all of the IIA package, either as the sole approach or in combination with other IA training and competency assessment packages.

Several units provided free-text comments about IA training and referred to efforts to “encourage” staff to access training in the absence of mandated training.

### IA practice audits

Overall 32% of respondents reported conducting IA practice audits at least annually; 36% reported no set frequency, and 32% reported not knowing the frequency of unit IA practice audits (Table 4). Sixty percent or nine of the 15 OUs in NHS organisations without MUs reported that there was no set frequency for IA practice audits. Sixty-one percent of units (*n*=100) reported having had an IA audit in the year prior to the survey. Thirty-one percent of AMU respondents (*n*=30) and 38% of those in FMUs (*n*=20) did not know the timing of the last audit, compared with only two respondents from OUs. A small number of respondents (2%, *n*=4) were not aware of an audit ever taking place, and four respondents from AMUs reported conducting monthly IA audits.

**Table 4:** Timing and content of IA practice audits by type of unit

	**AMU (*N*=101)**		**FMU (*N*=56)**		**OUs in NHS organisations without MUs (*N*=17)**		**Total (*N*=174)**		*p*-value*
	**n**	**%**	**n**	**%**	**n**	**%**	**n**	**%**	
**Frequency of IA practice audit**									
Every 6 months	23	22.8	11	19.6	3	20.0	37	21.5	
Annual	10	9.9	6	10.7	2	13.3	18	10.3	
Every other year	0	0.0	1	1.8	0	0.0	1	0.6	
No set frequency	33	32.7	19	33.9	9	60.0	61	35.5	
Don't know	35	34.7	19	33.9	1	6.7	55	32.0	0.385
*missing*	*0*		*0*		*2*		*2*		
**Year of last IA practice audit**									
2021-23	57	60.0	33	58.5	12	80.0	100	61.4	
2017-20	7	7.4	1	1.9	0	0.0	8	4.9	
Never had one	2	2.1	1	1.9	1	6.7	4	2.5	
Don't know	30	30.5	20	37.7	2	13.3	51	31.3	0.281
*missing*	*5*		*1*		*2*		*8*		
**Aspect of IA included in most recent audit**									
Admission / labour onset risk assessment **PLUS** frequency of auscultation in 1st stage **PLUS** 2nd stage	45	45.0	25	46.3	5	31.3	75	44.1	
Frequency of auscultation in 1st **PLUS** 2nd stage	11	11.0	6	11.1	2	12.5	19	11.2	
Other	5	5.0	2	3.7	4	25.0	11	6.5	
Don't know	39	39.0	21	38.9	5	31.3	65	38.3	0.243
*missing*	*1*		*2*		*1*		*4*		

^*^Fischer's exact test; missing excluded from test

Several respondents contributed free-text comments on the IA audit process, including about the opportunity for reflective monitoring/learning that IA audits offer.

*“From previous audits….we found that the frequency of auscultation in the second stage often means that the midwife is unable to maintain their documentation. Recording the fetal heart rates and maternal pulse on the partogram is vital for assessing fetal wellbeing, but doing this at least every 5 minutes for a prolonged period of time while facilitating birth is impossible. So we have introduced a second midwife for second stage. The role of the second midwife is to input the fetal & maternal heart rate recordings on to the partogram. The first midwife is then able to facilitate the “hands on” care such as timely auscultation and facilitating birth. This way the second midwife is able to have a helicopter view of any developing trends, similarities in maternal pulse or any fetal heart rate outside the normal range….We undertake a holistic review every hour with another midwife to ensure suitability to continue with intermittent auscultation”.* (AMU, England).

*“We undertake a peer review of IIA every three months. I have previously undertaken an observational audit of IIA techniques. This allows midwives to learn from good/bad IIA techniques and documentation*”. (AMU, England).

*“We audit fetal monitoring in labour (hospital births) every three months, however the notes that are audited are pulled at random and are audited regardless of fetal monitoring method, the number of cases with IA are relatively small in these audits*”. (OU in NHS organisation without MU, England).

*“The trust audit tool does not distinguish whether this is IA or continuous monitoring. Stats at present also reflect the Fetal Heart assessment when continuous monitoring is used, so it is not specific” *(OU in NHS organisation without MU, England)

When asked about the content of IA audits, 55% of all respondents reported that the most recent audit had covered multiple issues related to IA practice. However, a relatively high proportion of respondents in all three settings did not know the content of the most recent audit (Table 4).

### Availability of IA devices

The availability of equipment for IA monitoring varied by type of device but was broadly similar across different settings (Table 5). Pinard stethoscopes were almost universally available, followed by audio-only Doppler devices, and Dopplers with a number display. Half of AMUs the (*n*=51), 36% of FMUs (*n*=20), and 40% of the OUs in organisations without MUs, reported the availability of Doppler devices showing a fetal heart rate trace (FHRt). OUs in organisations without MUs were significantly more likely to report the availability of the CTG ultrasound head for use in IA than AMUs and FMUs were. Overall waterproof devices were reported to be *readily available* in 97% of the units (*N*=174). On average three different types of fetal monitoring devices were available for IA, and 8% of AMUs (*n*=8) and 5% of FMUs (*n*=3) reported having all five types of monitoring device we asked about (data not shown but details about the devices asked about are shown in Table S1).

**Table 5:** IA monitoring devices typically available by type of unit

	**AMU (*N*=101)**		**FMU (*N*=56)**		**OUs in NHS organisations without MUs (*N*=17)**		**Total (*N*=174)**		*p*-value*
	**n**	**%**	**n**	**%**	**n**	**%**	**n**	**%**	
**Availability of devices for IA**									
Waterproof devices (always)	99	98.0	54	96.4	15	88.2	168	96.6	0.131
Pinard stethoscopes	97	96.0	54	96.4	16	94.1	167	96.0	0.723
Doppler									
Audio only	91	90.1	48	85.7	13	76.5	152	87.4	0.232
With number display	66	65.4	38	67.9	11	64.7	115	66.1	0.971
With fetal heart rate trace	51	50.5	20	35.7	7	41.2	78	44.8	0.202
CTG ultrasound head	21	20.8	13	23.2	12	70.6	46	26.4	≤0.001
**Mean number of different devices available in unit**	3.2		3.1		3.4		3.2		

^*^Fischer's exact test used

A range of different combinations of monitoring devices were reported, the most common being Pinard stethoscopes, audio only Dopplers, and Dopplers with number displays, reported by just over a fifth of the MUs. Twenty-three percent of FMUs, and 16% of AMUs reported access to just two devices for IA - Pinard stethoscopes and audio-only Doppler devices. Pinard stethoscopes, audio-only Doppler devices, and Dopplers with a fetal heart rate trace (FHRt) were reported by 13% of AMUs compared with just 4% of FMUs (data not shown).

“*Intermittent auscultation is used daily in our unit. We use Dopplers with and without counters”. (AMU, Scotland).*

### Use of IA devices for fetal monitoring in midwifery led settings

Although Pinard stethoscopes were widely available for IA monitoring, they were less likely to be *typically* used in MUs. When used, Pinards tended to be used for the initial labour assessment (41%) rather than throughout labour (20%) (Table 6).

**Table 6:** IA monitoring device typically used during initial labour assessment and throughout labour by MU

	**AMU (*N*=101)**		**FMU (*N*=56)**		**Total (*N*=157)**		*p*-value*
	**n**	**%**	**n**	**%**	**n**	**%**	
**Device typically used for initial labour assessment**							
Pinard stethoscopes	38	37.6	27	48.2	65	41.4	0.237
Doppler							
Audio only	77	76.2	44	78.6	121	77.1	0.844
With number display	48	47.5	30	53.6	78	49.7	0.508
With fetal heart rate trace	37	36.6	11	19.6	48	30.6	0.031
CTG ultrasound head	3	3.0	1	1.8	4	2.6	1.00
**Device typically used throughout labour**							
Pinard Stethoscopes	17	16.8	14	25.0	31	19.8	0.295
Doppler							
Audio only	79	78.2	42	75.0	121	77.	0.695
Number display only	53	52.5	30	53.6	10	58.8	1.00
With FHRt	41	40.6	11	19.6	52	33.1	0.006
CTG ultrasound head	0	0.0	0	0.0	0	0.0	
**Admission CTG for low risk women**							
Sometimes (if clinically needed)	36	35.6**	10	17.9	28.7	46	
Never	65	64.4	46	82.1	70.7	111	0.032

^*^Fischer's exact test used

^**^includes 1 AMU that reported always conducting Admission CTG on low-risk women

**Figure 2:**
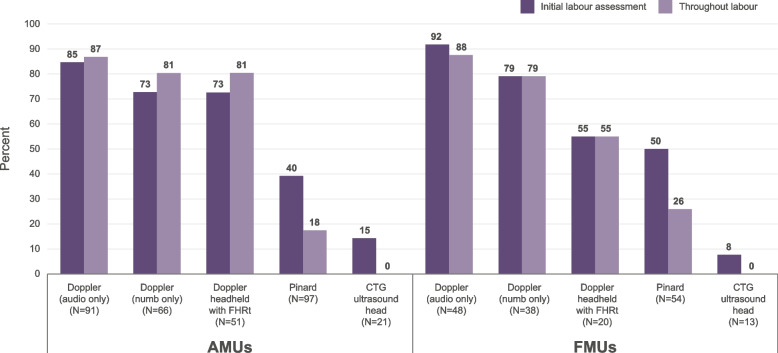
IA device typically used for initial labour assessment and throughout labour among MUs reporting IA device

The pattern of device use varied slightly by type of MU, with AMUs reporting greater use of Dopplers with an FHRt (37%) than FMUs did (20%). The availability of specific devices did not mean that they were typically used for IA (see Figure 2). While over 70% of AMUs reporting availability of Dopplers with an FHRt reported using them, just 55% of FMUs that reported their availability, reported using them (Figure 2). Among MUs that reported the availability of CTG for IA, only one FMU (out of 13), and three AMUs (out of 21) reported its use for IA monitoring.

As this respondent explained, availability of a device does not mean it is accessible or usable.

*“Pinards are readily available in every room including triage in the birth centre; however pinards and dopplers are difficult to locate on the labour ward. [There are] between one to three for the unit: one often in triage, one in theatre, one lost/not working. Pinards are not commonly used on the labour ward”. (AMU, England).*

Multiple monitoring devices in a variety of combinations were reported as typically used for IA. Three quarters of MUs reported that two or more devices were typically used, with the remainder reporting use of a single specific device. When a single device was typically used, audio-only Dopplers tended to dominate. Sixteen percent of AMUs and FMUs reported relying solely on audio-only Dopplers in the initial labour assessment, and this increased to a quarter of MUs throughout labour.

### Use of IA devices for fetal monitoring in obstetric units

All respondents were asked to select from a pre-assigned list of IA devices, all those typically used in OUs for initial labour assessment and throughout labour to monitor fetal wellbeing for health women with a straightforward pregnancy. The responses were broadly similar across different types of NHS organisation with or without MUs (Table 7). Pinard stethoscopes were typically used in 39% of organisations for initial labour assessment, decreasing to 12% during labour. Overall, audio-only Dopplers were the most common device used both for initial labour assessment and throughout labour, followed by Dopplers with a number display, and those with an FHRt**.**

**Table 7:** IA monitoring device use during initial labour assessment and throughout labour by type of OU

	**OUs in NHS organisations with MUs (*N*=99**±**)**^**a**^		**OUs in NHS organisations without MUs (*N*=17)**^**b**^		**Total (*N*=116)**		*p*-value*
	**n**	**%**	**n**	**%**	**n**	**%**	
**Device typically used for initial labour assessment**							
Pinard Stethoscopes	38	38.4	7	41.2	45	38.8	1.000
Doppler							
Audio only	80	80.1	11	64.7	91	78.4	0.316
Number display only	44^±^	44.4	9	52.9	53	45.7	0.714
With FHRt	34	34.3	7	41.2	41	35.3	0.843
CTG ultrasound head	21	21.2	6	35.3	27	23.3	0.439
CEFM^d^	29	29.3	1	5.9	30	25.9	0.068
Don't know	1	1.0^c^	0	0.0	1	0.9	<0.001
**Device typically used throughout labour**							
Pinard Stethoscopes	12	12.1	2	11.8	14	12.1	1.000
Doppler							
Audio only	76	76.8	12	70.6	88	75.0	0.622
Number display only	48	48.6	10	58.8	58	50.0	0.524
With FHRt	34	34.3	6	35.3	40	34.5	1.000
CTG ultrasound head	22	20.2	0	23.5	22	18.9	0.040
CEFM	20	22.2	4	0.0	24	20.7	0.751
Don't know	1	1.0	0	0.0	1	0.9	<0.001
**Admission CTG for low risk women**							
Always	6	6.0	0	0.0	6	5.2	
Sometimes (if clinically needed)	67	67.9	8	47.0	75	64.7	
Never	24	24.2	9	52.9	33	28.5	0.119

*Fischer's exact test

^a^Reported by midwifery unit respondents, prioritising responses from AMUs over FMUs. Responses from FMUs were included in the absence of an AMU response. N includes responses from 88 AMUs and 11 FMUs

^b^reported by respondents in OUs without midwifery units; ± 2 missing

^c^1 FMU reported not knowing

^d^ Continuous Electronic Fetal Monitoring

Respondents in MUs (reporting on the typical device used for monitoring in the OU in their NHS organisation) were more likely than respondents in OUs in organisations without MUs, to report that continuous electronic fetal monitoring (CEFM) was used for monitoring in healthy women with a straightforward pregnancy (Table 7).

### Admission CTG

We asked whether a short ‘admission’ CTG would be carried out for women who are healthy with a straightforward pregnancy. Responses varied by unit type (*p*<0.05) with 36% of AMUs (*n*=36) reporting that an admission CTG might be carried out if clinically needed, whereas 18% (*n*=10) of FMUs reported this (Table 6). A greater proportion of respondents from MUs (reporting on practice in OUs in their organisation) reported that admission CTGs would be carried out in the OU than respondents from OUs in NHS organisations without MUs (68% v. 47%; *p*=0.119) (Table 7).

### Home births

All respondents were asked about IA devices typically used for home births. Table 8 presents these data for NHS organisations with and without MUs. As with MUs (data shown in Table 6) Pinard use at home birth was more common for initial labour assessment than during labour, with some indications that Pinard use was more common at a home birth than for births in MUs (54% vs. 41%). Audio-only Doppler devices were the most typically used devices for IA at a home birth (77%).

**Table 8:** IA monitoring device used for home births and purchase of IA equipment by NHS organisation

	**NHS organisations with MUs (*N*=102)**^**a**^		**NHS organisations without MUs (*N*=17)**		**Total (*N*=119)**		
	**n**	**%**	**n**	**%**	**n**	**%**	*p*-value*
**Device typically used for initial labour assessment**							
Pinard stethoscopes	55	53.9	8	47.1	63	52.9	0.168
Doppler							
Audio only	81	79.4	10	58.8	91	76.5	0.049
Number display only	42	42.2	10	58.8	53	43.7	0.068
With FHRt	22	21.6	6	35.3	28	23.5	0.039
Don't know	8	7.8	0	0.0	8	6.7	0.098
**Device typically used throughout labour**							
Pinard stethoscopes	28	27.5	3	17.6	31	26.1	0.139
Doppler							
Audio only	78	76.5	10	58.8	88	74.0	0.044
Number display only	43	42.2	9	52.9	52	43.7	0.087
With FHRt	21	20.6	6	35.3	27	22.7	0.490
Don't know	9	8.8	0	0.0	9.0	7.6	0.107
**Type of Doppler device last purchased**							
Audio only Doppler only	44	43.6	7	43.8	51	43.6	
Doppler with number display only	14	13.9	4	25.0	18	15.4	
Doppler with FHRt	9	8.9	3	18.8	12	10.3	
Combination of two different types	22	21.9	1	6.3	23	19.7	
All three - Audio only Doppler, Doppler with number display, and Doppler with FHRt	3	3.0	0	0.0	3	2.6	
Don’t know	9	8.9	1	6.3	10	8.4	0.576
*missing*	*(1)*		*(1)*		*(2)*		

*Fischer’s exact test

^a^comprises 35 responses from FMUs and 67 from AMUs

### Purchase of Dopplers for maternity care

We asked all respondents about the type of Doppler devices last purchased for maternity care by their NHS organisation (Table 8). There was little variation between organisations with or without MUs. The most common device purchased was audio-only Doppler, purchased by 44% of NHS organisations.

### IA practice - “fresh ears” and counting methods

A buddy system for fresh ears for IA was reported by just under half of the MUs (48%), and 42% of OUs in organisations without midwifery-led units. (Table 9). Several units commented on the fresh ears approach.

*“I feel [IA] is being highly scrutinised in the birth centre at present. We have implemented lots of changes to aid documentation. Hourly 'buddy assessment' implementation is taking longer to embed than anticipated”* (AMU, England)

**Table 9:** Buddy system for IA in place, and required counting method by type of unit

	**AMU (*N*=101)**		**FMU (*N*=56)**		**OUs in NHS organisations without MUs (*N*=17)**		**Total (*N*=174)**		*p*-value*
	**n**	**%**	**n**	**%**	**n**	**%**	n	**%**	
**Buddy system for “fresh ears”**	49	48.5	27	48.2	7	41.7	83	47.7	0.9
**Required counting method**									
Watch only	43	42.6	19	33.9	3	17.7	65	37.4	
IIA** with 15 second block counting only	15	14.9	12	21.4	1	5.9	28	16.1	
IIA without 15 second block counting only	5	5.0	7	12.5	3	17.7	15	8.6	
No method required	13	12.9	2	3.6	6	35.3	21	12.1	
Watch, IIA without 15 second block counting	11	10.9	3	5.4	0	0.0	14	8.1	
Watch, IIA with 15 second block counting	7	6.9	5	8.9	0	0.0	12	6.9	
Other combination of counting method	7	6.9	8	14.3	4	23.5	19	10.3	
Percent using some form of IIA counting^a^	41	40.6	31	55.5	6	35.3	78	44.8	0.145

^*^Fischer’s exact test; ^a^Intelligent Intermittent Auscultation

All respondents were asked to select from a pre-assigned list (see Table S1), the methods they were required to use to count the fetal heart rate (Table 9). Over half of MU respondents reported that a single counting method was required – either counting against a watch, or the 15-second block counting recommended by the Intelligent Intermittent Auscultation (IIA) NHS e-learning package. Similar proportions of AMUs (25%, *n*=25) and FMUs (29%, *n*=16) reported two or more required counting methods in their units. Combining units listing only one method and those listing more than one method, the IIA approach, with or without the 15 second counting, was required for than half of the FMUs (56%), 41% of AMUs and 35% of OUs without midwifery units. The following quotes capture some of the variation in approaches to counting, and highlight some of the challenges associated with efforts to standardise practice.

*“The training day has shown us that there are variations in the counting methods used by our midwives, and they will frequently calculate different [Fetal Heart] values for the same FH demos. My observation is that this leads to variations in care management following FH assessment…”* (OU in NHS organisation without MU, England)

*“We are trying to embed the principles of IIA in our unit, however midwives are currently asked to follow the NICE guidance (at least every 15/5 minutes, immediately following a contraction, for at least 1 full minute), but are not told specifically what counting method to use. The plan…will be to put IIA block counting method into our Trust guidance….”* (OU in NHS organisation without MU, England).

*“Re counting method, staff can use any counting method they wish, but must count against a clock or watch*.” (AMU, England)

## Discussion

This study provides insight into aspects of current practice of IA in the UK. It was carried out primarily to inform the Listen2Baby study which aims to improve IA practice by providing evidence about the organisational and practice context for IA in the UK (https://www.npeu.ox.ac.uk/listen2baby). Given the very limited evidence concerning the best monitoring device, counting method, training and competency assessment for IA, these findings reveal a complex and varied practice landscape.

Most units reported using a combination of Pinard stethoscope and audio-only Doppler devices for IA, and approximately one-third typically used newer Doppler devices that also show a fetal heart trace. Most units reported that they had local guidance on IA in place; mandated annual IA training, and had access to a range of monitoring devices for IA as well as ready access to waterproof devices, but notably some did not. Our survey revealed significant variation in, and potential challenges for IA practice. A wide range of different IA training and assessment packages were in use; not all units mandated annual IA training and competency assessments for midwifery staff; and reporters’ knowledge of the timing and content of IA audits was low, with just a third of units reporting annual IA audits. At least six different methods for counting the fetal heart rate were reported, with 45% using some form of IIA counting. Just under half of units reported a buddy system for “fresh ears” in place for IA.

Against a background of little or no national guidance about some aspects of IA and little research evidence to support practice, most units reported that they had local IA guidance in place, but 16 reported that they did not, or that the guidance applied to women with midwifery led care only. In NHS organisations where midwifery led care is the default option for women at low risk of complications, local guidance for IA that applies only to women having midwifery led care, may be appropriate. However in 2019, 27 out of 151 NHS organisations which provided maternity care in the UK did not have midwifery led units [37]. Nationally, the proportion of women who give birth in midwifery led settings indicates that relatively high numbers of women who are at low risk of complications are admitted to OUs for labour and birth [19]. The extent to which IA is used for fetal monitoring in eligible women in OUs is unclear but will be explored in ethnographic work that is part of the Listen2Baby study. Research documenting the content of local IA guidance is also ongoing [38].

The RCOG 2001 guidelines on EFM advised that resources and time be made available to facilitate annual training and assessment in both IA & EFM, and that this should be reflected in local guidelines on fetal monitoring[15]. Since then, best practice guidance issued by NHS England as part of the Saving Babies Lives Care Bundle (SBLCB) [28–30] has recommended that all midwives undertake annual training and competency assessment in IA. Repeated national enquiries and quality improvement initiatives have recommended improved IA training, and called for more research about IA training and competency assessment [27]. While our survey found that most units had compulsory annual IA training (82%), just over half (56%) reported compulsory annual training and competency assessments, and 18% reported no compulsory training or that it was not required annually. We also found wide variation in the type of training provided, with ‘in-house’ training commonly reported. This may be an indicator of uncertainty about best practice, but may also reflect organisations trying to develop their own bespoke packages that address the elements required by the SBCLB . When a named training package was used, this was often the NHS e-learning for healthcare ‘Intelligent Intermittent Auscultation (IIA)’ training and competency assessment package; however, in approximately 15% of units this was combined with ‘in-house’ training, or the IIA training was used, but with ‘in-house’ assessment. There is no research evidence to recommend one method of IA or training package over another. A rigorous evaluation of training and assessment packages would support their use, or not, in practice. The authors of a recent systematic review, including over 60 studies about training in intrapartum CTG, reported that the evidence was often poor quality, with limited information about the optimal content and method of delivery [39].

National enquiries have also recommended that NHS organisations carry out regular audits of IA practice, including the frequency and timing of IA [23, 27]. In our survey, one-third of the respondents reported that audits of IA practice were at least annual, but overall, awareness of the occurrence, timing and content of IA audits was low among our respondents. It is possible that audits were taking place but that our respondents were unaware of them, suggesting that the results of any IA audits were not being effectively communicated to staff working in these units.

IA is typically carried out using a Doppler ultrasound device or a Pinard stethoscope, and a range of different Doppler devices are available, including some that show a trace of the fetal heart rate on a screen. It is also possible to use the ultrasound ‘head’ of a CTG machine for IA, although this was rarely reported by our respondents, and is discouraged in some units. There is little evidence to recommend one device over another, although one study included in a Cochrane review comparing the effectiveness of different devices for IA reported higher rates of caesarean birth for fetal distress among women monitored using a Doppler device, compared with routine monitoring with a Pinard [17, 40]. In addition to what we can infer from our survey results about typical use, which may be influenced by availability, there is no evidence about midwives’ preferences and the extent to which different devices support best practice.

The range and combination of different methods to count the fetal heart observed in this study, and that just under a half of NHS organisations had implemented the recommended buddy system for ‘fresh ears’ [28], points to further variation in terms of IA practice, both in areas where there is guidance and where guidance is lacking. The authors of a recent study of 303 UK midwifery students judged them knowledgeable about IA, but reported that some students lacked confidence in their ability to perform IA. Students attributed their lack of confidence to a lack of opportunity to observe and practice IA, and some perceived EFM as safer and more reassuring than IA[41] A nationally representative Norwegian survey revealed that CTG was used in half of all low-risk, straightforward births, and overall, CTG was used in 80% of births regardless of risk status. Exclusive IA monitoring was used in just 14% of births. The infrequent use of IA means less opportunity to practice the skills and techniques required, likely reducing its future use, especially when the Pinard device is used [2, 42]. Other likely contributing factors are a maternity service and staff under considerable pressure. Johanson *et al.* argued that ‘normal’ childbirth has become over medicalised and that the inappropriate use of EFM has increased worldwide. They suggest that higher rates of normal births are linked to beliefs about birth, implementation of evidence based practice, and team working[43]. Further investigation would be helpful to better understand why certain practices are followed or not, and the rationale behind these decisions in a clinical setting. These reasons are likely multifaceted and complex. The Listen2Baby ethnographic study will provide some of this evidence, but more research will also be needed to inform key questions including, for example, optimal methods for counting the fetal heart to ensure best outcomes.

In 2001 the RCOG recommended that future research was needed on the performance of different forms of IA, and how the performance of these modalities is affected by different frequencies of monitoring in comparison with EFM[15]. Potential variability in individual or institutional practice shown in this study needs to be considered when comparing maternal and fetal outcomes across all studies exploring fetal monitoring methods.

### Strengths and weaknesses

This was the first survey of IA practice in the UK. The overall response rate was high, particularly for MUs, where IA is most widely used, with at least one response from 119 (80%) of the 140 NHS Organisations contacted. While there was some evidence that AMUs were more likely to respond than FMUs, and there were varying responses from the different nations of the UK, the overall high response rate and the low levels of missing data, are strengths of our survey.

Using the UKMidSS infrastructure helped ensure a high response rate from ‘reporters’ who were already engaged with research. Most UKMidSS reporters are midwives with oversight of one or more MUs so they should be well placed to report on IA practice in their MU but may have less awareness of practices in other settings. This perhaps explains the observation that in NHS organisations with more than one responding MU we received some discordant responses about practice in OUs. This, coupled with the relatively lower response rate from OUs in organisations without midwifery led units, means that our findings about IA practice in OUs (and other organisation-level questions) are potentially less reliable than those about practice in MUs. Nevertheless, our survey provides evidence about IA practice where until now, there has been none.

## Conclusions

This national survey provides evidence of widespread variation in IA guidance, training, audit and practice across the UK. Evidence to inform IA best practice and standardise guidance is urgently needed.

## Supplementary Information

Supplementary Material 1.

Supplementary Material 2.

Supplementary Material 3.

## Data Availability

The dataset generated and analysed during the current study is not publicly available due to assurances given to participating units about confidentiality, but are available from the corresponding author on reasonable request.

## Abbreviations

AMU: Alongside midwifery unit
CEFM: Continuous Electronic Fetal Monitoring
CTG: Cardiotocography
FHRt: Fetal Heart Rate trace
FMU: Freestanding midwifery unit
IA: Intermittent Auscultation
IIA: Intelligent Intermittent Auscultation
K2: K2 Medical Systems
MLC: Midwifery led Care
MU: Midwifery led unit
NHS: National Health Service
NPEU: National Perinatal Epidemiology Unit
OU: Obstetric unit
PPI: Patient and public involvement
SBLCB: Saving Babies Lives Care Bundle
UK: United Kingdom
UKMidSS: United Kingdom Midwifery Study System
WHO: World Health Organisation
