# Supplementary material for: Intermittent auscultation fetal monitoring practice in different UK birth settings: a cross-sectional survey
Source: BMC Pregnancy Childbirth. 2025 Apr 14;25:446. doi: 10.1186/s12884-025-07514-2 (PMC11995465; doi:10.1186/s12884-025-07514-2)
Supplement: Supplementary file 1 — Supplementary Material 1. [file 12884_2025_7514_MOESM1_ESM.pdf]

Listen2Baby

Survey on intermittent auscultation practice in midwifery units

Intermittent auscultation in your midwifery unit

Intermittent auscultation devices

1. Which of the following are **available** for intermittent auscultation **in your midwifery unit**?
   Please select all that are available, even if not widely used
   01. Pinard stethoscope
   02. Hand held Doppler device without a number display (audio only)
   03. Hand held Doppler device with a number display
   04. Hand held Doppler device with a fetal heart rate tracing display
   05. CTG ultrasound head

Initial labour assessment

2. **In your midwifery unit,** which of the following are **typically used** for intermittent auscultation during **initial labour assessment**?
   Please select all that are typically used
   01. Pinard stethoscope
   02. Hand held Doppler device without a number display (audio only)
   03. Hand held Doppler device with a number display
   04. Hand held Doppler device with a fetal heart rate tracing display
   05. CTG ultrasound head

3. **In your midwifery unit,** is a short 'admission' CTG performed as part of the initial risk assessment for women with an uncomplicated pregnancy?
   Please select one only
   01. Always
   02. Sometimes / if clinically indicated
   03. Never

Ongoing labour monitoring

4. **In your midwifery unit,** which of the following devices are **typically used** for intermittent auscultation **throughout labour**?
   Please select all that are typically used
   01. Pinard stethoscope
   02. Hand held Doppler device without a number display (audio only)
   03. Hand held Doppler device with a number display
   04. Hand held Doppler device with a fetal heart rate tracing display
   05. CTG ultrasound head

5. **In your midwifery unit**, are **waterproof Doppler devices** readily available for intermittent auscultation during labour?
   Please select one only
   01. Always
   02. Sometimes
   03. Never

Intermittent auscultation practice

6. When undertaking intermittent auscultation, are midwives **in your midwifery unit** required to use any particular method to 'count' the fetal heart rate?

Please select all that apply

01. No, they can 'count' as they wish
02. Midwives are required to read the fetal heart rate off the Doppler
03. Midwives are required to count using a watch
04. Midwives are required to use the 'counting' method embedded in the NHS e-learning for healthcare 'Intelligent Intermittent Auscultation' programme (i.e. 15-second block counting)
05. Midwives are required to align their practice with the NHS e-learning for healthcare 'Intelligent Intermittent Auscultation' programme, but are not required to do 15-second block counting
06. Another counting method is used (please describe)

7. Do you use a 'buddy' system for 'fresh ears' for intermittent auscultation **in your midwifery unit**?

01. Yes
02. No

Intermittent auscultation training

8. Are midwives **in your midwifery unit** required to undertake mandatory training and assessment in intermittent auscultation?

01. No
02. Yes, training only
03. Yes, training and assessment

If yes, go to **8.1**

8.1. How often is the training required?

01. Every six months
02. Every year
03. Every other year
04. No set frequency

8.2. How often is the assessment required?

05. Every six months
06. Every year
07. Every other year
08. No set frequency
09. Not applicable

8.3. Which training package is mandated?

01. The NHS e-learning for healthcare 'Intelligent Intermittent Auscultation' programme **training and competency** assessment
02. The NHS e-learning for healthcare 'Intelligent Intermittent Auscultation' programme **training with 'in-house' competency** assessment
03. In-house training (please describe)
04. Other (please describe)

Audit of intermittent auscultation practice

9. How often is intermittent auscultation practice audited **in your midwifery unit**?

01. Every six months
02. Every year
03. Every other year
04. No set frequency
05. Don't know

10. When was the most recent audit of intermittent auscultation practice **in your midwifery unit**?

Month Year

11. Which aspects of intermittent auscultation were included in the most recent audit **in your midwifery unit**?
Please select all that apply
01. Admission / labour onset risk assessment
02. Frequency of auscultation in 1st stage
03. Frequency of auscultation in 2nd stage
04. Other (please describe)
05. Don't know

Intermittent auscultation devices used at home births

Initial labour assessment

12. Which of the following devices are **typically used** for intermittent auscultation during **initial labour assessment at a home birth**?
Please select all that are typically used
01. Pinard stethoscope
02. Hand held Doppler device without a number display (audio only)
03. Hand held Doppler device with a number display
04. Hand held Doppler device with a fetal heart rate tracing display
05. Don't know

Ongoing labour monitoring

13. Which of the following devices are **typically used** for intermittent auscultation **throughout labour at a home birth**?
Please select all that are typically used
01. Pinard stethoscope
02. Hand held Doppler device without a number display (audio only)
03. Hand held Doppler device with a number display
04. Hand held Doppler device with a fetal heart rate tracing display
05. Don't know

Intermittent auscultation in the obstetric unit/delivery suite

Initial labour assessment

14. Which of the following are **typically used** to assess fetal wellbeing during **initial labour assessment in the obstetric unit for a woman who is healthy with a straightforward pregnancy**?
Please select all that are typically used
01. Pinard stethoscope
02. Hand held Doppler device without a number display (audio only)
03. Hand held Doppler device with a number display

04. Hand held Doppler device with a fetal heart rate tracing display
05. CTG ultrasound head
06. Continuous electronic fetal monitoring
07. Don't know

15. **In the obstetric unit** is a short 'admission' CTG performed as part of the initial risk assessment for women who is healthy with a straightforward pregnancy?

Please select one only

01. Always
02. Sometimes / if clinically indicated
03. Never
04. Don't know

Ongoing labour monitoring

16. Which of the following are **typically used** for intermittent auscultation **throughout labour in the obstetric unit (labour ward) for a woman having straightforward labour**?

Please select all that are typically used

01. Pinard stethoscope
02. Hand held Doppler device without a number display (audio only)
03. Hand held Doppler device with a number display
04. Hand held Doppler device with a fetal heart rate tracing display
05. CTG ultrasound head
06. Continuous electronic fetal monitoring
07. Don't know

Purchase of fetal Doppler devices in your Trust/Health Board

17. When fetal Doppler devices for maternity care were last purchased in your Trust / Health Board, which type(s) were purchased?

Please indicate all purchased on the most recent order

01. Hand held Doppler device without a number display (audio only)
02. Hand held Doppler device with a number display
03. Hand held Doppler device with a fetal heart rate tracing display
04. Don't know

Local guidance about intermittent auscultation

18. Does your NHS Trust / Health Board have guidance about the use of intermittent auscultation in midwifery-led birth settings?

01. Yes – please upload/email guideline
02. No

19. Does your NHS Trust / Health Board have guidance about the use of intermittent auscultation in the obstetric unit?

01. Yes – please upload/email guideline
02. No

20. If there is anything else you would like to tell us about intermittent auscultation in your midwifery unit or elsewhere in your NHS Trust/Health Board please use the space below.
