# Supplementary material for: Intermittent auscultation fetal monitoring practice in different UK birth settings: a cross-sectional survey
Source: BMC Pregnancy Childbirth. 2025 Apr 14;25:446. doi: 10.1186/s12884-025-07514-2 (PMC11995465; doi:10.1186/s12884-025-07514-2)
Supplement: Supplementary file 2 — Supplementary Material 2. [file 12884_2025_7514_MOESM2_ESM.pdf]

Listen2Baby

# Survey on intermittent auscultation practice in obstetric units

## Intermittent auscultation in your obstetric unit (delivery suite)

### Intermittent auscultation devices

**1.** Which of the following are **available** for intermittent auscultation **in your obstetric unit**?
***Please select all that are available, even if not widely used***
01. Pinard stethoscope
02. Hand held Doppler device without a number display (audio only)
03. Hand held Doppler device with a number display
04. Hand held Doppler device with a fetal heart rate tracing display
05. CTG ultrasound head

**2.** **In your obstetric unit**, are **waterproof Doppler devices** readily available for intermittent auscultation during labour?
***Please select one only***
01. Always
02. Sometimes
03. Never

### Initial labour assessment

**3.** Which of the following are **typically used** to assess fetal wellbeing during **initial labour assessment in your obstetric unit for a woman who is healthy with a straightforward pregnancy**?
***Please select all that are typically used***
01. Pinard stethoscope
02. Hand held Doppler device without a number display (audio only)
03. Hand held Doppler device with a number display
04. Hand held Doppler device with a fetal heart rate tracing display
05. CTG ultrasound head
06. Continuous electronic fetal monitoring

**4.** **In your obstetric unit** is a short 'admission' CTG performed as part of the initial risk assessment for women who is healthy with a straightforward pregnancy?
***Please select one only***
01. Always
02. Sometimes / if clinically indicated
03. Never

## Ongoing labour monitoring

**5.** Which of the following are **typically used** for intermittent auscultation **throughout labour in your obstetric unit for a woman having straightforward labour**?

***Please select all that are typically used***

01. Pinard stethoscope
02. Hand held Doppler device without a number display (audio only)
03. Hand held Doppler device with a number display
04. Hand held Doppler device with a fetal heart rate tracing display
05. CTG ultrasound head
06. Continuous electronic fetal monitoring

## Intermittent auscultation practice

**6.** When undertaking intermittent auscultation, are midwives **in your obstetric unit** required to use any particular method to 'count' the fetal heart rate?

***Please select all that apply***

01. No, they can 'count' as they wish
02. Midwives are required to read the fetal heart rate off the Doppler
03. Midwives are required to count using a watch
04. Midwives are required to use the 'counting' method embedded in the NHS e-learning for healthcare 'Intelligent Intermittent Auscultation' programme (i.e. 15-second block counting)
05. Midwives are required to align their practice with the NHS e-learning for healthcare 'Intelligent Intermittent Auscultation' programme, but are not required to do 15-second block counting
06. Another counting method is used (please describe)

**7.** Do you use a 'buddy' system for 'fresh ears' for intermittent auscultation **in your obstetric unit**?

01. Yes
02. No

## Intermittent auscultation training

**8.** Are midwives **in your obstetric unit** required to undertake mandatory training and assessment in intermittent auscultation?

01. No
02. Yes, training only
03. Yes, training and assessment

If yes, go to **8.1**

**8.1.** How often is the training required?

01. Every six months
02. Every year
03. Every other year
04. No set frequency

**8.2.** How often is the assessment required?

05. Every six months
06. Every year
07. Every other year
08. No set frequency
09. Not applicable

**8.3.** Which training package is mandated?

01. The NHS e-learning for healthcare 'Intelligent Intermittent Auscultation' programme **training and competency** assessment
02. The NHS e-learning for healthcare 'Intelligent Intermittent Auscultation' programme **training with 'in-house' competency** assessment
03. In-house training (please describe)
04. Other (please describe)

### Audit of intermittent auscultation practice

**9.** How often is intermittent auscultation practice audited **in your obstetric unit**?

01. Every six months
02. Every year
03. Every other year
04. No set frequency
05. Don't know

**10.** When was the most recent audit of intermittent auscultation practice **in your obstetric unit**?

Month Year

**11.** Which aspects of intermittent auscultation were included in the most recent audit **in your obstetric unit**?

***Please select all that apply***

01. Admission / labour onset risk assessment
02. Frequency of auscultation in 1st stage
03. Frequency of auscultation in 2nd stage
04. Other (please describe)
05. Don't know

## Intermittent auscultation devices used at home births

### Initial labour assessment

**12.** Which of the following devices are **typically used** for intermittent auscultation during **initial labour assessment at a home birth**?

***Please select all that are typically used***

01. Pinard stethoscope
02. Hand held Doppler device without a number display (audio only)
03. Hand held Doppler device with a number display
04. Hand held Doppler device with a fetal heart rate tracing display
05. Don't know

### Ongoing labour monitoring

**13.** Which of the following devices are **typically used** for intermittent auscultation **throughout labour at a home birth**?

***Please select all that are typically used***

01. Pinard stethoscope
02. Hand held Doppler device without a number display (audio only)
03. Hand held Doppler device with a number display
04. Hand held Doppler device with a fetal heart rate tracing display

05. Don’t know

## Purchase of fetal Doppler devices in your Trust/Health Board

**14.** When fetal Doppler devices for maternity care were last purchased in your Trust / Health Board, which type(s) were purchased?

***Please indicate all purchased on the most recent order***

01. Hand held Doppler device without a number display (audio only)
02. Hand held Doppler device with a number display
03. Hand held Doppler device with a fetal heart rate tracing display
04. Don’t know

## Local guidance about intermittent auscultation

**15.** Does your NHS Trust / Health Board have guidance about the use of intermittent auscultation in the obstetric unit?

01. Yes – please upload/email guideline
02. No

**16.** Does your NHS Trust / Health Board have guidance about the use of intermittent auscultation at home births?

01. Yes – please upload/email guideline
02. No

**17.** If there is anything else you would like to tell us about intermittent auscultation in your NHS Trust/Health Board please use the space below.
