# Supplementary material for: Intermittent auscultation fetal monitoring practice in different UK birth settings: a cross-sectional survey
Source: BMC Pregnancy Childbirth. 2025 Apr 14;25:446. doi: 10.1186/s12884-025-07514-2 (PMC11995465; doi:10.1186/s12884-025-07514-2)
Supplement: Supplementary file 3 — Supplementary Material 3. [file 12884_2025_7514_MOESM3_ESM.docx]

| SUPPLEMENTARY TABLE  Table S1 Topics and response categories covered in survey |
| --- |
| **NHS Organisation has local IA guidance in place** |
| - For midwifery-led settings (MU and for home births): yes / no |
| - For OU: yes / no |
| **IA practice, availability and use of IA devices in unit (MU or OU)** |
| - IA training and competency assessment regularity: (i) Every 6 months; (ii) annually; (iii) every other year; (iv) no set frequency; (v) don’t know. |
| - Mandated training package: (i) NHS e-learning for healthcare IIA programme training and competency assessment; (ii) NHS e-learning for healthcare IIA programme training with in-house competency assessment; (iii) in-house training (specify/describe); (iv) other (describe). |
| - IA practice audit regularity: (i) Every 6 months; (ii) annually; (iii) every other year; (iv) no set frequency; (v) don’t know. |
| - Date of most recent IA practice audit |
| - Aspect of IA practice include in most recent audit *(tick all that apply):* (i) admission / labour onset risk assessment; (ii) frequency of auscultation in 1^st^ stage; (iii) frequency of auscultation in 2^nd^ stage; (iv) other (describe); (v) don’t know. |
| - IA device(s) **availability** in your unit, *(tick all that apply)*: (i) pinard stethoscope; (ii) hand held Doppler (audio only); (iii) hand held Doppler (number display); (iv) hand held Doppler (fetal heart rate tracing (FHRt) display); (v) CTG ultrasound head. |
| - IA device(s) **typically used** for **initial labour assessments** in your unit *(tick all that apply)*: (i) pinard stethoscope; (ii) hand held Doppler (audio only); (iii) hand held Doppler (number display); (iv) hand held Doppler (fetal heart rate tracing (FHRt) display); (v) CTG ultrasound head. |
| - IA device(s) **typically used** **throughout labour** in your unit *(tick all that apply)*: (i) pinard stethoscope; (ii) hand held Doppler (audio only); (iii) hand held Doppler (number display); (iv) hand held Doppler (fetal heart rate tracing (FHRt) display); (v) CTG ultrasound head. |
| - Short admission CTG performed as part of initial risk assessment (Never, sometimes (if clinically required), always). |
| - Required counting method when undertaking IA: (i) none prescribed; (ii) read the fetal heart rate from the Doppler; (iii) use of watch; (iv) 15 second block counting embedded in NHS e-learning for healthcare “intelligent intermittent auscultation (IIA)” programme; (v) NHS e-learning for healthcare “IIA” programme without 15-second block counting; (vi) other (specify). |
| - Buddy system used for fresh ears: yes/no. |
| **Use of IA devices during home births in your NHS organisation** |
| - IA device(s) typically used for **initial labour assessments in home births** (tick all that apply): (i) pinard stethoscope; (ii) hand held Doppler (audio only); (iii) hand held Doppler (number display); (iv) hand held Doppler (fetal heart rate tracing (FHRt) display); (v) CTG ultrasound head; (vi) Don’t know. |
| - IA device(s) typically used throughout **labour during home births** (tick all that apply): (i) pinard stethoscope; (ii) hand held Doppler (audio only); (iii) hand held Doppler (number display); (iv) hand held Doppler (fetal heart rate tracing (FHRt) display); (v) CTG ultrasound head; ); (vi) Don’t know. |
| **Use of IA devices in OU(s) in your NHS organisation** |
| - IA device(s) typically used for **initial labour assessments** in the OU *(tick all that apply)*: (i) pinard stethoscope; (ii) hand held Doppler (audio only); (iii) hand held Doppler (number display); (iv) hand held Doppler (fetal heart rate tracing (FHRt) display); (v) CTG ultrasound head; (vi) short admission CTG performed as part of initial risk assessment; vi) Don’t know. |
| - IA device(s) typically used **throughout labour** in the OU *(tick all that apply)*: (i) pinard stethoscope; (ii) hand held Doppler (audio only); (iii) hand held Doppler (number display); (iv) hand held Doppler (fetal heart rate tracing (FHRt) display); (v) CTG ultrasound head); (vi) Don’t know. |
| **Purchase of IA devices in your NHS organisation** |
| - IA device(s) **last purchased** by Trust / Health Board (tick all that apply): (i) hand held Doppler (audio only); (ii) hand held Doppler (number display); (iii) hand held Doppler (fetal heart rate tracing (FHRt) display); (iv) Don’t know. |
